# Comparison of AAPM Addendum to TG‐51, IAEA TRS‐398, and JSMP 12: Calibration of photon beams in water

**DOI:** 10.1002/acm2.12159

**Published:** 2017-08-03

**Authors:** Naoki Kinoshita, Hiroshi Oguchi, Yasuhiro Nishimoto, Toshiki Adachi, Hiroki Shioura, Hirohiko Kimura, Kunio Doi

**Affiliations:** ^1^ Department of Radiological and Medical Laboratory Sciences Nagoya University Graduate School of Medicine Nagoya‐shi Aichi‐ken Japan; ^2^ Radiological Center University of Fukui Hospital Yoshida‐gun Fukui‐ken Japan; ^3^ Department of Radiology University of Fukui Hospital Yoshida‐gun Fukui‐ken Japan; ^4^ Department of Radiology University of Chicago Chicago IL USA; ^5^ Gunma Prefectural College of Health Sciences Maebashi‐shi Gunma‐ken Japan

**Keywords:** absorbed dose to water, Addendum to TG‐51 protocol, IAEA TRS‐398 protocol, JSMP 12 protocol, photon clinical reference dosimetry

## Abstract

The American Association of Physicists in Medicine (AAPM) Working Group on TG‐51 published an Addendum to the AAPM's TG‐51 protocol (Addendum to TG‐51) in 2014, and the Japan Society of Medical Physics (JSMP) published a new dosimetry protocol JSMP 12 in 2012. In this study, we compared the absorbed dose to water determined at the reference depth for high‐energy photon beams following the recommendations given in AAPM TG‐51 and the Addendum to TG‐51, IAEA TRS‐398, and JSMP 12. This study was performed using measurements with flattened photon beams with nominal energies of 6 and 10 MV. Three widely used ionization chambers with different compositions, Exradin A12, PTW 30013, and IBA FC65‐P, were employed. Fully corrected charge readings obtained for the three chambers according to AAPM TG‐51 and the Addendum to TG‐51, which included the correction for the radiation beam profile (*P*_rp_), showed variations of 0.2% and 0.3% at 6 and 10 MV, respectively, from the readings corresponding to IAEA TRS‐398 and JSMP 12. The values for the beam quality conversion factor *k*_Q_ obtained according to the three protocols agreed within 0.5%; the only exception was a 0.6% difference between the results obtained at 10 MV for Exradin A12 according to IAEA TRS‐398 and AAPM TG‐51 and the Addendum to TG‐51. Consequently, the values for the absorbed dose to water obtained for the three protocols agreed within 0.4%; the only exception was a 0.6% difference between the values obtained at 10 MV for PTW 30013 according to AAPM TG‐51 and the Addendum to TG‐51, and JSMP 12. While the difference in the absorbed dose to water determined by the three protocols depends on the *k*_Q_ and *P*_rp_ values, the absorbed dose to water obtained according to the three protocols agrees within the relative uncertainties for the three protocols.

## 1 INTRODUCTION

The incidence and death rates of cancer have recently been increasing worldwide,^1^ highlighting the need for effective cancer treatment methods. Radiation therapy is considered an important step for effective cancer treatment. In radiation therapy, a high‐accuracy dose is required to be delivered to the patient to achieve a favorable clinical outcome.^2^, ^3^ Therefore, high‐energy radiation sources are commonly calibrated under certain reference conditions according to a protocol based on the ionization chambers, which are calibrated using the standards for the absorbed dose to water.

During the last two decades, the International Atomic Energy Agency (IAEA), the American Association of Physicists in Medicine (AAPM), and other national organizations from various countries have published clinical reference dosimetry protocols for external beam radiation therapy using high‐energy photon and electron beams. The formalism and dosimetry procedures specified in publications such as those of AAPM Task Group 51 (TG‐51),^4^ IAEA Technical Report Series No. 398 (TRS‐398),^5^ Institute of Physical Sciences in Medicine (IPSM 1990),^6^ and DIN 6800‐2,^7^ are based on the use of an ionization chamber with a ^60^Co absorbed dose to water calibration factor, $N_{D,w}^{60_{C}o}$, and a beam quality conversion factor, *k*_Q_, for the radiotherapy beam.

These standards for absorbed dose to water can reduce the uncertainty in the dosimetry of radiotherapy beams^5^ and can provide a more robust system of primary standards than air‐kerma‐based standards. Further, since the publication of AAPM TG‐51 and IAEA TRS‐398, Monte Carlo (MC) simulation methods^8^, ^9^ have been developed and accurately benchmarked^10^, ^11^, ^12^ for the calculations of detailed chamber geometries.^13^, ^14^, ^15^ Moreover, studies in current literature have provided ionization chamber perturbation correction factors^15^, ^16^, ^17^ and beam quality conversion factors.^14^, ^15^, ^18^ Although the AAPM TG‐51 provided *k*_Q_ factors for only cylindrical chambers, which represented the majority of reference chambers available at the time of publication, more than 30 different designs for ionization chambers have recently become available. Therefore, the AAPM Working Group on TG‐51 published an AAPM Addendum to TG‐51^19^ and the Japan Society of Medical Physics (JSMP) published a new protocol for JSMP (JSMP 12).^20^ Owing to the new accurate values of the *k*_Q_ factor provided in the AAPM Addendum to TG‐51^19^ and JSMP 12,^20^ the uncertainties in the absorbed dose to water would be smaller than those in the previous reference dosimetry protocols. However, while AAPM TG‐51,^4^ IAEA TRS‐398,^5^ and other protocols have been discussed extensively in literature,^21^, ^22^, ^23^, ^24^, ^25^ particularly with respect to the advantages and disadvantages of the recommended photon beam quality indices,^26^, ^27^, ^28^, ^29^ the IAEA TRS‐398,^5^ AAPM TG‐51 and Addendum to TG‐51,^19^ and JSMP 12^20^ protocols have not yet been compared. The differences that may exist among these protocols could have important consequences for dosimetric evaluation; therefore, in this study, we compared the absorbed dose to water determined for high‐energy photon beams according to AAPM TG‐51 and the Addendum to TG‐51, JSMP 12, and IAEA TRS‐398.

## 2 METHODS

### 2.A Materials

Three reference‐class cylindrical‐type ionization chambers (typically, Farmer‐type chambers) were investigated. The characteristics of the chambers are given in Table 1. The Exradin A12 (Standard Imaging, Middleton, WI, USA) and PTW 30013 (PTW, Freiburg, Germany) chamber types are widely used as evidenced by the frequency of their calibration by three accredited dosimetry calibration laboratories in the United States,^30^ and therefore, they are representative chambers for comparing the three protocols. The absorbed dose to water calibration factor $N_{D,w}^{60_{C}o}$ for the three chambers was provided by a Japanese secondary standard dosimetry laboratory (Association for Nuclear Technology in Medicine Japan). The calibration factor $N_{D,w}^{60_{C}o}$ and voltage for the Exradin A12, PTW 30013, and FC65‐P (IBA Dosimetry GmBH, Schwarzenbruck, Germany) chambers were 4.902 × 10^−2^ Gy/nC with a voltage of −299 V, 5.33 × 10^−2^ Gy/nC with a voltage of −300 V, and 4.858 × 10^−2^ Gy/nC with a voltage of −300 V, respectively. Then, the Exradin A12, PTW 30013, and FC65‐P chambers were connected to a SuperMAX electrometer (Standard Imaging, Middleton, WI, USA), a RAMTEC 1000 plus electrometer (Toyo Medic, Tokyo, Japan), and a Keithley 35040 Therapy Dosimeter electrometer (Keithley Instruments Inc., Cleveland, OH, USA), respectively.

**Table 1:** Characteristics of the three chambers used

	Sensitive cavity			Wall		Central electrode
Chamber type	Volume (cm^3^)	Length (mm)	Radius (mm)	Material	Thickness (mm)	Material
Exradin A12	0.65	24.8	3.05	C552	0.5	C552
PTW 30013	0.6	23.0	3.05	PMMA/Gr	0.335/0.09	Al
IBA FC65‐P	0.65	23.1	3.1	POM	0.4	Al

C‐552: air equivalent plastic; PMMA: polymethylmethacrylate; Gr: graphite; POM: polyoxymethylene; Al: aluminum.

All the chambers were loaned by the vendors and other institutions, and had to be returned within a short period of time. Therefore, it was not possible to examine the long‐term stability for the three chambers. Alternatively, the stability of the three ion chambers was analyzed using long‐term $N_{D,w}^{60_{C}o}$ data. The change in the $N_{D,w}^{60_{C}o}$ values over the typical recalibration period of 2 yr was 0.2%, 0.2%, and 0.1% for Exradin A12, PTW 30013, and FC65‐P, respectively. The Addendum to TG‐51 states that the change in the calibration factor over the typical recalibration period of 2 yr should be less than a 0.3%.^19^ The three ionization chambers met the minimum requirement of megavoltage photon‐beam dosimetry.

Measurements for the absorbed dose to water dosimetry and beam quality were performed using a Siemens Artiste linear accelerator (Siemens AG, Erlangen, Germany) using flattened photon beams with energies of 6 MV and 10 MV. Because a modern linear accelerator was used, the short‐term repeatability of the linear accelerator when delivering a series of fixed monitor‐unit runs was assumed to be less than 0.05%.^19^, ^31^ Dosimetry for the absorbed dose to water and measurements of beam quality were performed using a WP1D water phantom (IBA Dosimetry, Schwarzenbruck, Germany). Given the uncertainties in setting the origin for the water phantom software and the movement distance of the chambers from the origin to the reference depth of 10 cm, the uncertainty in the positioning of the chamber at the reference depth (coverage factor *k* = 1) was estimated at approximately 0.4 mm.

### 2.B Beam quality for the three protocols

For IAEA TRS‐398 and JSMP 12, it was recommended to use the tissue‐phantom ratio (TPR_20,10_) as the beam quality index in order to choose the appropriate beam quality conversion factors, whereas AAPM TG‐51 and the Addendum to TG‐51 used the percent depth dose obtained at 10 cm depth, *%dd*(10)_x_, excluding the electron contamination effect.

Several procedures were used for calculating the beam quality index. For 6 MV, *%dd*(10)_x_ was obtained as equal to the measured *%dd*(10). For photon energies of 10 MV or above, the electrons from the accelerator head may considerably affect the dose at *d*_max_ and, hence, may reduce the measured value of *%dd*(10). Therefore, we used a 1‐mm lead filter to remove the contaminating electrons and replace them with the better‐known contamination from lead. For the photon energy of 10 MV, *%dd*(10)_Pb_ was measured by placing the lead filter at a distance of 46 cm from the water surface and using the equations in the AAPM TG‐51 protocol for removing the signal from the electrons generated in lead based on percent depth dose (PDD) measurements. The factor, *TPR*_*20,10*_, was given by the ratio of ionization on the beam central axis at depths of 20 and 10 cm in water, as obtained with a field size of 10 × 10 cm^2^ at the ionization chamber and a constant source‐to‐detector distance of 100 cm.

### 2.C Determination of absorbed dose by the three protocols

The absorbed dose to water was determined on the basis of the recommendations in the three protocols. The comparison of the reference conditions for the determination of the absorbed dose to water and formalism according to the three protocols are shown in Tables 2 and 3, respectively. Because detailed information about the three protocols can be found in the protocol documents, only the major differences relevant to this study are described below.

**Table 2:** Reference conditions for determination of absorbed dose to water for the three protocols

	AAPM TG‐51 and Addendum to TG‐51	JSMP 12	IAEA TRS‐398
Phantom material	Water		
Beam quality	*%dd*(10)_x_	*TPR*_*20,10*_	*TPR*_*20,10*_
Chamber type	Cylindrical	Farmer	Cylindrical
Reference depth	10 g cm^−2^	10 g cm^−2^	*TPR*_*20,10*_ <0.7, 10 g cm^−2^(or 5 g cm^−2^) *TPR*_*20,10*_ ≥ 0.7, 10 g cm^−2^
Measurement point of the chamber		The center of the cavity	
SSD/SAD		100 cm	
Field size		10 × 10 cm^2^	

**Table 3:** Formalism for determination of absorbed dose to water for the three protocols

	AAPM TG‐51 and Addendum to TG‐51	JSMP 12, and IAEA TRS‐398
Formalism	*Mk*_*Q*_ $N_{D,w}^{60_{C}o}$	*M*_*Q*_ $N_{D,w,Q_{0}}k_{Q,Q_{0}}$
Fully corrected charge reading	*M*_raw_ *P*_TP_ *P*_ion_ *P*_pol_ *P*_elec_ *P*_leak_ *P*_rp_	*M*_*raw*_ *k*_*TP*_ *k*_*elec*_ *k*_*pol*_ *k*_*s*_

*M* and *M*_Q_: fully corrected charge reading; $N_{D,w}^{60_{C}o}$ and $N_{D,w,\;Q_{0}}$: absorbed dose to water calibration factor for the ^60^Co beam; *k*_*Q*_ and $k_{Q,\;Q_{0}}$: beam quality conversion factor; *M*_raw_: uncorrected charge reading; *P*_TP_ and *k*_TP_: temperature and pressure correction factor; *P*_ion_ and *k*_s_: correction for incomplete ion collection efficiency; *P*_pol_ and *k*_pol_: correction for any polarity effects; *P*_elec_ and *k*_elec_: correction for the electrometer; *P*_leak_: correction for any contribution to the measured reading that is not due to the ionization released by the radiation beam; *P*_rp_: correction for considering any off‐axis variation in the intensity profile of the radiation field over the sensitive volume of the ionization chamber.

In the following Section 2.C text, we use the notations consistent with the AAPM TG‐51 and Addendum to TG‐51 protocols. In IAEA TRS‐398 and JSMP 12, several notations are comparatively different from those in AAPM TG‐51 and the Addendum to TG‐51; however, all quantities can essentially be translated to the quantities in the TRS‐398 formalisms without loss of meaning. The absorbed dose to water $D_{w}^{Q}$ in a photon beam with beam quality *Q* is obtained from the following equation.

(1) $$D_{w}^{Q}=Mk_{Q}N_{D,w}^{60_{Co}},$$

Where

*M*: fully corrected charge reading from an ionization chamber,

*k*_*Q*_: beam quality conversion factor,

$N_{D,w}^{60_{C}o}$: absorbed dose to water calibration factor for the ^60^Co beam.

(2) $$M=M_{\mathrm{raw}}P_{\mathrm{TP}}P_{\mathrm{ion}}P_{\mathrm{pol}}P_{\mathrm{elec}}P_{\mathrm{leak}}P_{\mathrm{rp}},$$

Where

*M*_raw_: uncorrected charge reading,

*P*_TP_: temperature and pressure correction factor,

*P*_ion_: correction for incomplete ion collection efficiency,

*P*_pol_: correction for any polarity effects,

*P*_*elec*_: correction for the electrometer,

*P*_leak_: correction for any contribution to the measured reading that is not due to the ionization released by the radiation beam,

*P*_rp_: correction for considering any off‐axis variation in the intensity profile of the radiation field over the sensitive volume of the ionization chamber.

These correction factors were experimentally determined according to the three protocols. The values for *P*_leak_ and *P*_rp_ are not included in IAEA TRS‐398 and JSMP 12.

For all three protocols, the reference conditions for measurements of absorbed dose to water included a 100 cm source–surface distance (SSD) or source‐axis distance (SAD), 10 × 10 cm^2^ field size at a 100 cm SSD or 100 cm SAD, and a 10 cm depth in water with IAEA TRS‐398 allowing a 5 cm depth if *TPR*_*20,10*_ is less than 0.7. For this study, measurements of the absorbed dose to water for the three protocols were performed with an SAD of 100 cm, a depth of 10 cm, and a field size of 10 × 10 cm^2^ at the ionization chamber with the angle for the gantry and the collimator set at 0°. Therefore, in this study, the uncorrected charge readings, *M*_raw_, at the center of the chamber cavity for all protocols were considered to be the same to reduce the experimental uncertainty in *M*_raw_ corresponding to an average of at least five readings. The relative humidity was always within 20% to 60% and the humidity correction was not necessary according to the recommendation of the three protocols as long as the relative humidity was in the range of 20% to 80%.^4^, ^5^, ^19^, ^20^, ^32^

### 2.D Quantification of uncertainties for the three protocols

In this study, photon beam calibration was performed only once with each ionization chamber. Because the same set of readings was used to determine the beam calibrations regardless of the protocol applied, the experimental uncertainties, such as the temperature and pressure correction factor, the short‐term repeatability of the linear accelerator, setup of field size, SSD/SAD, and chamber depth, were the same for the three protocols. The correction factor for the polarity effect for the three protocols was determined using the same equation. Similarly, as the same set of readings at each voltage was used to determine the correction for the polarity effects, the uncertainty in the polarity effect of the correction factor was the same for the three protocols. In addition, the same $N_{D,w}^{60_{C}o}$ factor was used across the three protocols for a given ionization chamber. The uncertainties in the $N_{D,w}^{60_{C}o}$ factor were also the same for the three protocols. In contrast, the user‐dependent parts, such as the assignment of the beam quality conversion factor, determination of the correction for incomplete ion collection efficiency, *P*_leak_, *P*_rp_, and beam quality conversion factor, were performed using different procedures and had different values for the three protocols. In terms of the beam quality conversion factor, the three protocols provided the uncertainty. Therefore, we estimated the uncertainties in the user‐dependent parts, namely, the assignment of the beam quality conversion factor, determination of the correction for incomplete ion collection efficiency, *P*_leak_, and *P*_rp_, according to the ISO Guide to the Expression of Uncertainty in Measurement^33^ in order to evaluate the small differences observed among the three protocols.

## 3 RESULTS

### 3.A Correction factors for charge readings by an ionization chamber for the three protocols

The correction factors for the charge readings obtained for the three ionization chambers using the three protocols are shown in Table 4. Because the temperature‐pressure correction and polarity correction factors for the three protocols were calculated using the same formula, these correction factors had the same values in all three protocols. In addition, because the correction factors for ion collection efficiency by IAEA TRS‐398 and JSMP 12 were calculated using the same formula, the correction factors had the same value in these two protocols. The ion collection efficiencies, *P*_ion_ and *k*_s_, as determined by the three protocols, agreed within 0.1%. The *P*_rp_ factors according to the Addendum to TG‐51 were 1.002 at 6 MV and 1.003 at 10 MV.

**Table 4:** Correction factors for charge readings from three ionization chambers obtained by the three protocols

		AAPM TG‐51 and Addendum to TG‐51						JSMP 12				IAEA TRS‐398			
Nominal MV	Chamber	*P*_TP_	*P*_ion_	*P*_pol_	*P*_elec_	*P*_leak_	*P*_rp_	*k*_TP_	*k*_s_	*k*_pol_	*k*_elec_	*k*_TP_	*k*_s_	*k*_pol_	*k*_elec_
6	Exradin A12	0.990	1.0019	1.000	1	1	1.002	0.990	1.0018	1.000	1	0.990	1.0018	1.000	1
	PTW 30013	1.005	1.0024	0.999	1	1	1.002	1.005	1.0023	0.999	1	1.005	1.0023	0.999	1
	IBA FC65‐P	0.994	1.0013	1.001	1	1	1.002	0.994	1.0013	1.001	1	0.994	1.0013	1.001	1
10	Exradin A12	0.990	1.0042	0.999	1	1	1.003	0.990	1.0040	0.999	1	0.990	1.0040	0.999	1
	PTW 30013	1.009	1.0042	1.000	1	1	1.003	1.009	1.0039	1.000	1	1.009	1.0039	1.000	1
	IBA FC65‐P	0.994	1.0055	0.999	1	1	1.003	0.994	1.0053	0.999	1	0.994	1.0053	0.999	1

The fully corrected charge readings for the three ionization chambers obtained according to the three protocols are shown in Table 5 with the fully corrected charge readings obtained according to TRS‐398 and JSMP 12 showing identical values. The fluctuations in the uncorrected charge readings for each ionization chamber amounted to approximately ± 0.1%. The differences between the values of the fully corrected charge readings obtained using the AAPM TG‐51 and Addendum to TG‐51 protocols and the IAEA TRS‐398 and JSMP 12 protocols were found to be 0.2% and 0.3% at 6 and 10 MV, respectively.

**Table 5:** Fully corrected charge readings (nC) from three ionization chambers obtained according to the three protocols

Chamber	Protocol	6 MV	Difference (%)	10 MV	Difference (%)
Exradin A12	AAPM TG‐51 and Addendum to TG‐51	16.05	–	17.67	–
	JSMP 12	16.01	−0.2	17.62	−0.3
	IAEA TRS‐398	16.01	−0.2	17.62	−0.3
PTW 30013	AAPM TG‐51 and Addendum to TG‐51	14.689	–	16.090	–
	JSMP 12	14.656	−0.2	16.043	−0.3
	IAEA TRS‐398	14.656	−0.2	16.043	−0.3
IBA FC65‐P	AAPM TG‐51 and Addendum to TG‐51	16.238	–	17.978	–
	JSMP 12	16.202	−0.2	17.925	−0.3
	IAEA TRS‐398	16.202	−0.2	17.925	−0.3

Difference (%) = (Fully corrected charge readings for JSMP 12 or IAEA TRS‐398−Fully corrected charge readings for AAPM TG‐51 and Addendum to TG‐51)/Fully corrected charge readings for AAPM TG‐51 and Addendum to TG‐51.

### 3.B Beam quality conversion factor for the three protocols

The beam quality conversion factors obtained according to the three protocols are shown in Table 6. These factors obtained according to the three protocols agreed within 0.6%, 0.4%, and 0.3%, for Exradin A12, PTW 30013, and IBA FC65‐P, respectively.

**Table 6:** Beam quality conversion factors for the three protocols

		6 MV		10 MV	
Chamber	Protocol	*TPR*_*20,10*_ = 0.671 %*dd*(10)_x_ = 67.0	Difference (%)	*TPR*_*20,10*_ = 0.740 %*dd*(10)_x_ = 74.5	Difference (%)
Exradin A12	Addendum to TG‐51	0.992	–	0.980	–
	JSMP 12	0.997	0.5	0.984	0.4
	IAEA TRS‐398	0.995	0.3	0.986	0.6
PTW 30013	Addendum to TG‐51	0.991	–	0.979	–
	JSMP 12	0.990	−0.1	0.976	−0.3
	IAEA TRS‐398	0.992	0.1	0.980	0.1
IBA FC65‐P	Addendum to TG‐51	0.991	–	0.979	–
	JSMP 12	0.992	0.1	0.979	−0.1
	IAEA TRS‐398	0.994	0.3	0.981	0.2

Difference (%) = (Beam quality conversion factors for JSMP 12 or IAEA TRS‐398−Beam quality factors for Addendum to TG‐51)/Beam quality conversion factors for Addendum to TG‐51.

### 3.C Determining absorbed dose by the three protocols

The absorbed doses to water obtained according to the three protocols are shown in Table 7. The measured dose with the Exradin A12 chamber determined according to AAPM TG‐51 and the Addendum to TG‐51 was 0.1% and 0.3% lower than that determined according to IAEA TRS‐398, at 6 and 10 MV, respectively. The measured dose for the PTW 30013 chamber with AAPM TG‐51 and the Addendum to TG‐51 was 0.15% and 0.2% higher than that with TRS‐398. The measured dose with the IBA FC65‐P chamber obtained according to AAPM TG‐51 and the Addendum to TG‐51 was 0.03% lower than that obtained according to TRS‐398 at 6 MV, whereas the dose obtained according to AAPM TG‐51 and the Addendum to TG‐51 was 0.1% higher than that obtained according to TRS‐398 at 10 MV.

**Table 7:** Absorbed doses to water (Gy) according to the three protocols

Chamber	Protocol	6 MV	Difference (%)	10 MV	Difference (%)
Exradin A12	AAPM TG‐51 and Addendum to TG‐51	0.780	–	0.849	–
	JSMP 12	0.783	0.3	0.850	0.1
	IAEA TRS‐398	0.781	0.1	0.851	0.3
PTW 30013	AAPM TG‐51 and Addendum to TG‐51	0.776	–	0.840	–
	JSMP 12	0.773	−0.3	0.835	−0.6
	IAEA TRS‐398	0.775	−0.1	0.838	−0.2
IBA FC65‐P	AAPM TG‐51 and Addendum to TG‐51	0.782	–	0.855	–
	JSMP 12	0.781	−0.1	0.852	−0.4
	IAEA TRS‐398	0.782	0.03	0.854	−0.1

Difference (%) = (Absorbed doses for JSMP 12 or IAEA TRS‐398 − Absorbed doses for AAPM TG‐51 and Addendum to TG‐51)/Absorbed doses for AAPM TG‐51 and Addendum to TG‐51.

The measured dose with the Exradin A12 chamber determined with JSMP 12 was 0.2% higher than that with TRS‐398 at 6 MV, whereas the measured dose with JSMP 12 was 0.2% lower than that with TRS‐398 at 10 MV. The measured dose with the PTW 30013 chamber determined according to JSMP 12 was 0.2% and 0.4% lower than that determined according to TRS‐398, at 6 and 10 MV, respectively. The measured dose with the IBA FC65‐P chamber determined with JSMP 12 was 0.2% and 0.3% lower than that with TRS‐398, at 6 and 10 MV, respectively.

### 3.D Quantification of uncertainties

The ion recombination factors for the three protocols were calculated using a two‐voltage method. The relative standard uncertainties (*k* = 1) in *k*_s_ for JSMP 12 and IAEA TRS‐398 and P_ion_ for AAPM TG‐51 were 0.09% and 0.07% at most, respectively.

The relative standard uncertainties in TPR_20,10_ for JSMP 12 and IAEA TRS‐398 were approximately 0.3% (*k* = 1). The uncertainty was from two main sources. The first source was the components associated with the setup for the reference conditions and their measurements. The second was the use of ionization ratios in place of ratios of absorbed dose in the determination of *TPR*_*20,10*._ 34 When determining the beam quality conversion factor with interpolation, the relative standard uncertainties were 0.05% (*k* = 1) at the maximum.

The relative standard uncertainties in *%dd*(10)_x_ (*k* = 1) were 0.30% and 0.42% for 6 MV and 10 MV, respectively. The uncertainty in *%dd*(10)_x_ for 6 MV was only considered in the determination of *%dd*(10)_x_, such as in the setup for the reference conditions and their measurements. In contrast, the uncertainty in *%dd*(10)_x_ for 10 MV was considered due to the equation used for converting *%dd*(10)_Pb_ into *%dd*(10)_x_ 23 as well as in the determination of *%dd*(10)_Pb_. When determining the beam quality conversion factor with the polynomial fits in the Addendum to TG‐51, the relative standard uncertainties (*k* = 1) were 0.05% at the maximum, as with IAEA TRS‐398 and JSMP 12.

The leakage currents were measured with all the equipment used in this study, but with no beam. The leakage currents were below the 0.1% level of the chamber reading. Thus, we assumed *P*_leak_ = 1.000 with an associated relative uncertainty (*k* = 1) of 0.1%.^19^ The radiation dose profiles under the reference conditions at a 10 cm depth were determined from a simple 1‐D beam profile measurement using a two‐dimensional detector array MapCHECK2 (Sun Nuclear Corporation, Melbourne, FL, USA). The *P*_rp_ factors were calculated from the measured beam profiles. The relative uncertainty for the *P*_rp_ was assumed to be 0.05%^19^ (*k* = 1).

## 4 DISCUSSION

For the three protocols investigated in this study, a major difference in the fully corrected charge readings, *M*, was observed due to the variation in *P*_rp_, as shown in Table 4. The fully corrected charge readings determined according to AAPM TG‐51 and the Addendum to TG‐51, which included *P*_rp_, were 0.2% and 0.3% higher at 6 MV and 10 MV, respectively, than those determined according to IAEA TRS‐398 and JSMP 12. Traditional flattened beams can usually produce uniform beams for a 10 × 10 cm^2^ field, and therefore, the *P*_rp_ value could be close to 1.000. In this study, because of non‐uniformities in the beam, the variation in *P*_rp_ will be a major contributor to the observed discrepancy in *M* for the three protocols. The same is true for “horns” in a beam profile.^35^

The *k*_Q_ factors for the three protocols were determined by different methods, giving rise to the possibility that different *k*_Q_ values will be obtained by these methods. The factors for the Addendum to TG‐51 were determined using full MC calculations incorporating detailed information about the chamber to better reflect the true chamber geometry.^14^, ^19^ In contrast, the result for IAEA TRS‐398 was based on a semi‐analytic approach that did not consider all details of the chamber geometry.^5^, ^36^ Perturbation correction factors used in the expression for *k*_Q_ for JSMP 12 were obtained from MC calculations, with the exception of a wall correction factor derived by semi‐analytical expressions.^20^ Therefore, the difference in the *k*_Q_ factors for the three protocols appears due to the difference between the perturbation factors obtained by a semi‐analytic approach and MC calculations. Several studies^14^, ^15^, ^16^, ^36^ have compared individual perturbation correction factors obtained using MC calculations for cylindrical chambers with those obtained using a semi‐analytic approach, such as that used in IAEA TRS‐398 and AAPM TG‐51. For a ^60^Co beam, the value of the replacement factor derived from MC calculations by Wang and Rogers^17^ was 0.5% higher than the AAPM TG‐51 value and approximately 1% higher than the IAEA TRS‐398 value. Buckley and Rogers^16^ and Muir and Rogers^14^ showed that the values of the wall correction factors derived from MC calculations for a wall material comprising PMMA, such as that found in PTW 30013, a wall material comprising C‐552, such as that found in Exradin A12, and a wall material comprising POM, such as that found in IBA FC65‐P, differed by −0.2%–0.4% from the AAPM TG‐51 value. However, in this study, the *k*_Q_ factors for the three protocols agreed within a relative uncertainty of 1.0% in the *k*_Q_ values for IAEA TRS‐398 and JSMP 12. In particular, the *k*_Q_ values for PTW 30013 and IBA FC65‐P obtained according to the three protocols agreed well within an uncertainty of 0.4% in the *k*_Q_ values for the Addendum to TG‐51.

Because the same $N_{D,w}^{60_{C}o}$ factor was used across all of the three protocols for a given ionization chamber, the major discrepancy in the absorbed dose to water values obtained for these protocols was due to the difference in the *P*_rp_ and *k*_Q_ values. As stated in the previous section, the obtained values of absorbed dose to water for the three protocols agreed within 0.4%, with the exception of a 0.6% difference at 10 MV between AAPM TG‐51 and the Addendum to TG‐51 and JSMP 12 obtained for PTW 30013, indicating that there is a good agreement within the relative uncertainty for the absorbed dose to water given by the Addendum to TG‐51 (Table 2 situation (i) of Ref. 19), IAEA TRS‐398, and JSMP 12. The *P*_rp_ may depend on the particular linear accelerator used. Furthermore, the values of the *k*_Q_ factors should be chamber‐specific. This study is concerned with a comparison of the absorbed dose to water as determined for high‐energy photon beams according to the three protocols using only the three cylindrical chambers, because of which the potential difference in the absorbed dose to water for the three protocols using a wide range of ionization chambers and linear accelerators remains unknown. Future work is necessary for the comparison of the absorbed dose to water as determined for high‐energy photon beams according to the three protocols using a wide range of ionization chambers and linear accelerators.

## 5 CONCLUSION

The absorbed dose to water for the three protocols using the three ionization chambers showed good agreement within the relative uncertainty in the absorbed dose to water given by the three protocols. Because of the use of cylindrical ionization chambers with the same $N_{D,w}^{60_{C}o}$, the major discrepancy between the obtained values of the absorbed dose to water for the three protocols occurred due to the difference in the *P*_rp_ and the *k*_Q_ values. The *P*_rp_ and the *k*_Q_ values may depend on the linear accelerator and cylindrical ionization chamber used, respectively. Thus, the absorbed dose to water determined for high‐energy photon beams according to the three protocols would change depending on the linear accelerator and the cylindrical ionization chamber used in the experiment.

## CONFLICT OF INTEREST

The authors declare no conflict of interest.
